# A Pin Retained in the Body Nineteen Years and Then Discharged

**Published:** 1840-11

**Authors:** 


### A PIN RETAINED IN PHE BODY NINETEEN YEARS AND THEN DISCHARGED.

We are indebted to Dr. Darius Maxon, of Gallipolis, Ohio, for the following fact, which presented itself in his practice a few weeks since.

Nineteen years ago a lady swallowed a pin, which was followed by symptoms of aggravated dyspepsia. For several days before it was discharged she was affected with a pain in her left side for which the Doctor applied an antimonial plaster, that produced pimples, through one of which the pin made its exit. A slight discharge of bloody serum followed. The pin was considerably coated and its head removed to its middle. D.

